# Exploring Community Co-Creation in Tree Planting and Heat-Related Health Interventions: A Qualitative Study

**DOI:** 10.3390/ijerph22060896

**Published:** 2025-06-04

**Authors:** Olivia J. Keenan, Aalayna R. Green, Alexander R. Young, Sarah R. Young, Daniel S. W. Katz, David L. Miller, Wenna Xi, Fiona Lo, Evelyn Ortiz, Glenn McMillan, Curtis L. Archer, Arnab K. Ghosh

**Affiliations:** 1 Division of General Internal Medicine, Department of Medicine, Weill Cornell Medicine, 420 E 70th St, New York, NY 10065, USA; 2 Department of Natural Resources & the Environment, Cornell University, 111 Fernow Hall, Ithaca, NY 14853, USA; arg267@cornell.edu; 3 Earth Systems Research Center, University of New Hampshire, 8 College Road, Durham, NH 03824, USA; 4 School of Integrative Plant Science, Cornell University, 306 Tower Rd., Ithaca, NY 14853, USA; 5 Division of Biostatistics, Department of Population Health Sciences, Weill Cornell Medicine, 425 E 61st St, New York, NY 10065, USA; 6 Environmental Defense Fund, 1875 Connecticut Ave NW Ste 600, Washington, DC 20009, USA; 7 Improve Chronic Disease Outcomes Through Multi-Level and Multi-Generational Approaches Unifying Novel Interventions and Training for Health Equity (COMMUNITY) Center Collaborative, 622 W 168th St, New York, NY 10032, USA; 8 Columbia University Center for Community Health, 610 W 130th St., New York, NY 10027, USA; 9 Harlem Community Development Corporation, 163 W. 125th St, 17th Floor, New York, NY 10027, USA

**Keywords:** environmental justice, heat-related health, community co-creation, tree planting, extreme heat, transdisciplinary, qualitative research

## Abstract

Climate-amplified extreme heat events are particularly dangerous for city dwellers. Nature-based solutions such as urban greening may serve as an effective preventative strategy against extreme heat. Driven by historical injustices such as redlining, disadvantaged communities often face limited green space and a heightened risk of vulnerability to extreme heat in urban environments. This paper investigates community engagement strategies for heat-vulnerable community participation in urban greening research as a part of a broader transdisciplinary environmental research praxis focused on multistakeholder co-creation. We conducted semi-structured interviews with community leaders in heat-vulnerable neighborhoods in New York City to explore community co-creation in the design and implementation of tree planting, and compared these themes with interviews with urban tree professionals and other community groups. Overall, the participants agreed on broad themes of environmental justice, intergenerational engagement, community building, and socioecological relationships, although community leaders differed in both a greater emphasis of experiential knowledge and reduced focus on volunteer community stewardship. The findings inform our research process and associated community engagement, including building online resources and addressing community-specific concerns during the research process. We conclude by recommending future steps for facilitating multistakeholder conversations to build inclusive and equitable urban greening heat-adaptive strategies.

## 1. Introduction

Anthropogenic climate change is resulting in more frequent and intense heat waves [1,2], increasing the threat of heat-related morbidity and mortality [3,4,5]. Extreme heat events are the deadliest extreme weather event [6], worsening and causing health conditions such as respiratory disease, cardiovascular disease, digestive illness, psychological disorders, and adverse pregnancy outcomes [7,8]. The threat of extreme heat is a particular concern in cities due to the urban heat island effect, which leads to higher temperatures in urban areas due to a combination of factors such as reduced vegetation cover, increased anthropogenic heat sources, and greater heat storage in the building materials of urban infrastructure [9,10].

A well-known solution to cooling is through greening, or the expansion of forests, parks, gardens, natural areas, and other vegetation cover [11,12]. Cities are therefore key sites for designing urban greening interventions in response to worsening extreme heat [13]. One such example is New York City (NYC); NYC’s urban green spaces play a large role in climate mitigation [14,15], and urban greening is expected to be an integral climate mitigation strategy in response to heat and public health risks [11,16,17]. In fact, in 2023, the NYC Council passed Resolutions 1065/1066 (2023 Local Law 148), which codified the City’s intention to increase the tree canopy cover from 22% to 30% through an Urban Forest Plan [18]. This resolution further recognizes that, despite recent efforts to expand tree cover, urban greening benefits are often unequally distributed across communities, with many racially and socio-economically marginalized communities receiving fewer benefits. Notably, NYC’s inequity in green infrastructure is an example of a nationwide issue: areas with a higher density of racially and economically marginalized groups have significantly less access to tree cover benefits and more intense urban heat island effects across the United States [19,20,21,22].

The successful and sustainable implementation of urban greening within disadvantaged communities requires an environmental justice approach to achieve its ends. Environmental justice is the equitable distribution of environmental harms and benefits; in the case of urban greening, it is the equitable distribution of tree cover regardless of age, race, class, or neighborhood [23]. The tenets of environmental justice often focus on three aspects: recognitional justice (i.e., who are the subjects of injustice?), distributive justice (i.e., how is justice/injustice distributed?), and procedural justice (i.e., how are decisions made?) [23,24,25]. Recognitional justice acknowledges who is most affected by environmental harms (e.g., heat vulnerability) and who has the least access to environmental benefits (e.g., tree cover), while distributive justice aims to ameliorate these injustices by addressing the historical and systemic factors that drive distributions of injustice [26,27]. Procedural justice is the meaningful inclusion and participation of communities in environmentally just solutions (i.e., co-creation) [23].

Equitable urban greening requires a collaborative approach in order to ensure community voices are reflected and uplifted in urban greening decision-making (i.e., procedural justice), making urban greening itself a successful enterprise [28]. Failure to include community voices in urban greening campaigns has the potential to drive displacement of communities due to green gentrification, opposition to tree planting, or a lack of community maintenance and care of trees, all of which hinders the success of a greening project [29,30,31,32,33]. To address these challenges, examples within the literature describe “co-creative” practices, including participatory planning and model validation by centering on the community’s voice [28,34]. Community involvement seeks to challenge traditional research paradigms, and involve intergenerational, racial, gender, and socioeconomic considerations of engagement, whereby the community is given the same access to power and prioritization in urban greening solutions as, in this particular case, researchers, environmental professionals, and planners [35,36,37,38]. Thus, knowledge and insights from multiple stakeholders, informed by an intersectional and transdisciplinary approach that addresses multiple identity axes and hierarchical systems of domination [39,40,41], can elucidate possible solutions to the causes of environmental inequality. Through this type of co-creation, communities provide insight and knowledge that are different from and invaluable to environmental professionals and planners, extending beyond disciplinary boundaries and improving the practicality and usability of urban greening [42]. Often, in scientific research, this type of knowledge is referred to as embodied knowledge or experiential knowledge, which is the knowledge and information gained from an individual’s lived experiences [43,44]. Despite this, the literature has described the lack of procedural justice and community co-creation in urban greening research and decision-making [23,45].

Concerns about environmental justice and the role and importance of community involvement become more important as urban greening gains popularity in cities around the world, and in the US. In the US, the Inflation Reduction Act has invested over $1 billion in U.S. urban forestry programs [46]. As urban greening campaigns inevitably increase in the age of climate change, policymakers and urban greening researchers will need to understand the values and lived experiences of the affected communities in order to effectively build resilience. This multistakeholder, equity-focused work calls for transdisciplinary research approaches, which require transcending disciplinary boundaries and incorporating community-engaged scholarship [28,47]. This differs from interdisciplinary approaches, which integrate different disciplines, yet often fail to communicate new knowledge back within specific discipline/stakeholder structures; by contrast, transdisciplinary research approaches “attempt to form their own intellectual space” through a “novel paradigm of collaboration”, including centering community knowledge [28].

This paper represents the beginning of our research team’s ongoing transdisciplinary process as we ask the question: how can researchers effectively co-create urban greening research and design alongside communities? We explore this question through semi-structured interviews with community leaders in NYC and compare themes with interviews from urban forest professionals and other community groups, to inform our own research process, as well as the processes of others undertaking similar environmental justice or community health interventions. Informed by the literature, the goal of this work is to adhere closely to an inclusive research methodology that incorporates and sustains the procedural and distributive aspects of environmental justice throughout our larger research project, which is to employ the urban forest as a heat-adaptive tool in the era of climate change.

## 2. Materials and Methods

### 2.1. Overview of Participants and Interviews

**Figure 1:**
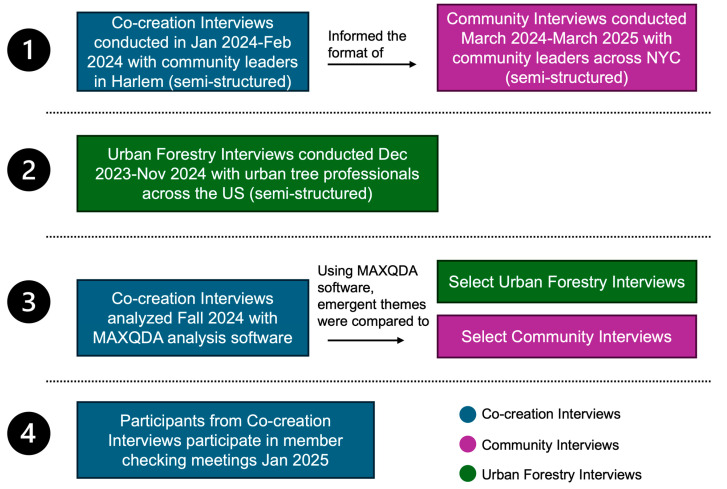
Graphic of interview process.

We conducted three types of semi-structured interviews in a sequential series that informed our co-creation process (summarized in Figure 1). These three series were as follows: (1) Co-Creation Interviews with community leaders whose knowledge informed our subsequent interview schedule, thematic analysis, and review of our findings; (2) Community Interviews with community leaders from other communities; and (3) Urban Forestry Interviews with urban tree professionals.

**Figure 2:**
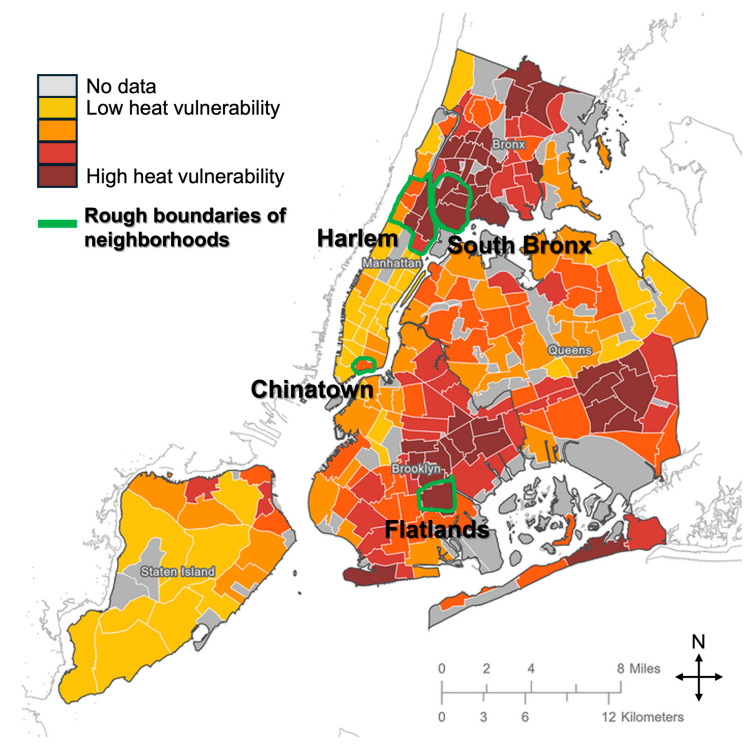
Map of New York City heat-vulnerable areas and participant neighborhoods. Adapted from the interactive heat vulnerability index developed by the NYC Department of Health and Mental Hygiene’s Bureau of Environmental Surveillance and Policy [48,50].

The Co-Creation Interviews were the first interviews administered by the research team and conducted with community leaders in Harlem—a heat-vulnerable neighborhood in NYC, as determined by the interactive heat vulnerability index of NYC (Figure 2; darker orange and red areas indicate heat vulnerability) [48]. The heat vulnerability index determines neighborhoods whose residents are more at risk for dying due to extreme heat based on factors such as temperature, air conditioning, green space, and median income [48]. The boundaries of “Harlem” include the sub-neighborhoods Morningside Heights, Manhattanville, Central Harlem, East Harlem, Hamilton Heights, and Sugar Hill. Community leaders were determined based on involvement in community advisory boards, community-based organizations, small-business ownership, community health work, and other broad means of community leadership. The main goal of these first interviews was to explore the ways to ensure that we, as researchers, involved the community’s voice from the outset of our research, but also act as a source of advisement and check on community co-creation and engagement throughout the research process. We took this step because equitable and transdisciplinary urban greening research approaches emphasize the importance of co-production of knowledge through collaborative research [42,49]. An excerpt from our interview script’s introductory prompt describes the goals of the Co-Creation Interviews in the following way:

*“Co-creating research ensures voices of participants are heard and valued to the same extent as scientific knowledge. For us, this looks like building questions for the interviews and focus groups together, communicating about how we can analyze the data from interviews and focus groups, and working together on presenting findings. The questions below will guide us throughout the research process.”*

Six total Co-Creation Interviews were conducted, with one participant per interview. Six interviews were conducted because saturation was reached after three interviews. After the Co-Creation Interviews, a coding scheme was produced with emergent themes.

Next, informed by the Co-Creation Interviews, the Community Interviews and Urban Forestry Interviews were administered by the research team using separate interview formats. Community Interview participants were different community leaders from heat-vulnerable neighborhoods across NYC (Figure 2) [48], and the goal of the interviews was to explore community perceptions around trees as a solution to heat. Interviews were conducted with participants in the South Bronx, Harlem, Flatlands, and Chinatown (Figure 2). Urban Forestry Interview participants were urban tree professionals across the US, and the goal of the interviews was to explore the practical considerations of tree planting and management related to cooling. As of March 2025, 14 Community Interviews and 34 Urban Forestry Interviews were conducted. For this study, five interviews of each were randomly chosen because of the availability of finalized interviews at the time of this analysis (all 14 Community Interviews and 34 Urban Forestry Interviews were not finalized until months after the analysis for this manuscript). The coding scheme derived from the Co-Creation Interviews was applied to the Community and Urban Forestry Interviews to compare and contrast the emergent themes. We did this because juxtaposing findings between stakeholders is an essential part of transdisciplinary work [28].

Finally, we validated our findings through member-checking meetings with the original Co-Creation Interview participants. These meetings allowed participants to provide meaningful feedback on our findings. Participants were also offered co-authorship opportunities.

See Table 1 for a description of the participants and interviews and Figure 1 for a schematic of the process.

### 2.2. Co-Creation Interviews and Inductive Coding

#### 2.2.1. Co-Creation Interview Process

First, our research team established relationships with an external Community Advisory Board (CAB), a group that consists of community liaisons in NYC already embedded in community health research. We first presented our research plan to CAB members in September 2023; then, in a follow-up December 2023 meeting, we asked interested CAB members if they would be willing to meet with us to help us build out our research co-creation plan. We connected with five interested participants from the CAB and one additional participant from a CAB member’s suggestion.

Co-Creation Interviews were conducted from January through February 2024. Interviews were 1 h long on Zoom. Participants were compensated with a $50 VISA gift card for their time in the study. Interviews were audio-recorded and then transcribed by Ubiqus, a professional transcription service [51]. The semi-structured interview schedule can be found in Supplementary File S1. Interviews were semi-structured because we had set questions to guide initial conversation but often asked follow-up questions based on participant responses.

#### 2.2.2. Inductive Coding Analysis

The interviews were coded using an inductive coding strategy. In this case, we first applied an explorative, inductive approach to the data; one coder (OJK) open coded (i.e., inductive) the first three Co-Creation Interview transcripts. Then, a second coder (ARG) sorted these codes into emergent themes, creating a new coding scheme with “axial codes”, or higher-level categories. The first coder (OJK) then used the new coding scheme to recode the first three Co-Creation Interviews, and the last three Co-Creation Interviews. The second coder (ARG) then finalized the five emergent themes described in the Results Section 3. The coders utilized MaxQDA qualitative analysis software version 24.6.0 [52].

### 2.3. Other Interviews and Deductive Coding

#### 2.3.1. Community and Urban Forestry Interviews

After conducting interviews and analyzing themes from our Co-Creation Interviews, we sought, through snowballing, connections with other community leaders in NYC, focused again on heat-vulnerable neighborhoods (Chinatown, Manhattan; Flatlands, Brooklyn; Harlem, Manhattan; and South Bronx). Heat-vulnerable neighborhoods were determined according to the NYC heat vulnerability index developed by the NYC Department of Health and Mental Hygiene’s Bureau of Environmental Surveillance and Policy [48,50]. To mitigate bias in responses, participants were chosen based on either geographic or occupational diversity. For example, participants were recruited with different occupations than the initial Co-Creation participants (e.g., business owner, environmental justice organizer, etc.) and/or with different neighborhood experience.

These interviews specifically focused on exploring community perspectives around trees and heat. The semi-structured interview schedule can be found in Supplementary File S1. Study participants were recruited through snowballing (i.e., having previous participants invite others to the study) from the Co-Creation Interviews as well as other professional connections. Community interviews were conducted from March 2024 to March 2025. Interviews were 1 h long and conducted on Zoom or in person. Participants were compensated with a $50 VISA gift card for their time. All interviews were audio-recorded and transcribed by Ubiqus or an AI transcription service (Otter.ai) [51,53].

Separately, from December 2023 to November 2024, our study team conducted interviews with urban tree professionals across the United States. These interviews explored the concerns of urban tree professionals with using trees to combat heat. Urban tree professionals included city arborists, urban forest researchers, and urban forest stewardship managers. The semi-structured interview questions can be found in the Supplementary File S1. Interviews were 1 h long and conducted on Zoom. For these interviews, occasionally more than one participant was present, as indicated by the ten participants across the five chosen interviews. All interviews were audio-recorded and transcribed by Ubiqus or an AI transcription service (Otter.ai) [51,53].

#### 2.3.2. Deductive Coding Analysis

We coded the Urban Forestry and Community Interviews using the codebook derived from the Co-Creation Interviews (i.e., a priori, deductive coding). One coder (OJK) coded the ten transcripts and summarized the coded quotes for each theme to deduce similarities and differences between interview types. A second coder (ARG) independently reviewed the coding, and the two coders reached consensus over email. MaxQDA qualitative analysis software was used for this analysis as well [52].

### 2.4. Member Checking and Research Co-Creation

In December 2024, we invited the original participants from the Co-Creation Interviews to review the qualitative findings. The goal of the meetings was to provide open discussion on our results (e.g., member checking). Member checking is a methodological practice in qualitative research that allows participants to provide feedback on research findings [54]. In our research process, member checking was one part of the co-creation process, whereby community members are engaged throughout the research process. Additionally, at the conclusion of the meeting, we also offered co-authorship opportunities for this paper with transparent expectations for co-authorship. The purpose of this step was to ensure our research involved continued avenues of co-creation, particularly in the presentation of research results.

## 3. Results

### 3.1. Inductive Data Analysis

Our inductive coding of the six Co-Creation Interviews with community leaders resulted in five themes that answered the question, how can researchers effectively co-create urban greening research and design alongside communities??

1. Environmental justice and health equity are interlinked and at the heart of this research;
2. Religious and spiritual communities are important touchpoints in urban greening research;
3. Intergenerational engagement ensures intergenerational inclusion;
4. Human–environment relationships vary by individual and community;
5. Community building makes co-creation and knowledge sharing between communities and researchers possible.

**Figure 3:**
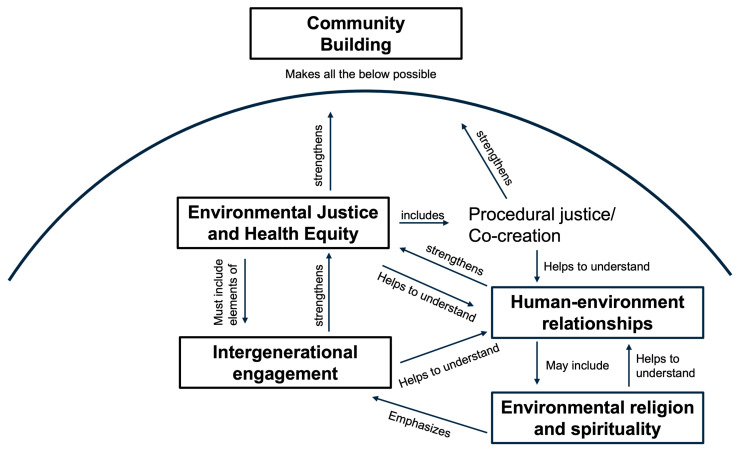
Conceptual diagram of themes.

See Table 2 for a summary of each theme and Figure 3 for a visual representation of themes.

#### 3.1.1. Inductive Analysis: Environmental Justice and Health Equity Are Interlinked and at the Heart of This Research

Participants placed a heavy emphasis on environmental justice- and health equity-focused research, stressing that environmental justice and health equity are inextricably linked. Participants further noted that their communities wanted to be healthy, yet environmental injustices such as heavy air pollution or lack of green space prevent this. They specifically reported that environmental hazards also predominantly affect medically underserved communities who historically have not been included in medical research or environmental justice intervention processes. Participants therefore emphasized the importance of including impacted communities in research. In order to have an environmentally just health intervention, they argued that researchers need to embody environmental justice practices (procedural justice and co-creation, intergenerational community engagement, long-lasting relationships, transparency, and education). One participant emphasized the consequences of not including community members in intervention design, noting that communities may no longer identify with their lived environment when community co-creation is absent:

*“So, that way, most importantly, in my context, we’re not gentrifying and creating something that people can no longer identify with but being inclusive, by involving not just myself, but the little kid, the grandmother…”* (Co-Creation Interview, Participant #5)

Participants also noted that researchers need to be clear on the benefits to health and the environment because communities deserve evidence-based information, just like scientific publications or public policy, but shared in a way that is transparent and understandable. Additionally, interventions should have clearly defined measures of success that matter to the community:

*“It* [urban greening implementation] *looks good, and what not, but… didn’t really do much. So, no, we want to know that the measures have an impact, we believe a positive impact, on the community. We want to see that. We want to know that. How do you measure that?”* (Co-Creation Interview, Participant #3)

The participants urged that a positive impact on the community was essential for any public health intervention. In this way, embodying all the tenets of environmental justice was and is crucial to addressing the link between environmental injustice and health inequity.

#### 3.1.2. Inductive Analysis: Religious and Spiritual Communities Are Important Touchpoints in Urban Greening Research

Two participants discussed how communities of faith have special connections with the environment and provided ideas for engaging with particularly Christian audiences. Through stories in the Bible, the Christian faith teaches reciprocity with the Earth, and therefore faith communities feel a divine responsibility to be good stewards:

*“So, from my perspective, the importance of the spiritual part involving the living trees or how God gave us this* [world]*, we should care for it and how it will in return care for us, because that’s really what the theology of sociology is under. It’s a thought that if we take care of what God has given us, then in return, it will take care of us, which is really what I think a lot of scientists are trying to say.” *(Co-Creation Interview, Participant #5)

Furthermore, both emphasized the fact that faith communities are familiar with waiting for future benefits. A forester may plant a small acorn seed, and diligently care for the sapling, but must wait to reap the benefits of a tall oak’s shade for decades. Similarly, a person of faith must plant the seeds of their faith and nurture and grow themselves throughout their life, ultimately for salvation or a perfect reunion with God at the end of life. Participants discussed how this parallel way of thinking can be utilized by researchers in teaching about stewardship and care for the environment, with an emphasis on the intergenerational aspects of urban greening specifically, but also environmental justice in general:

*“…when you talk about trying to share with people that there are future effects that a tree will have when it grows, the faith community is so understanding of that because that’s exactly one of the major components of what faith is. Believing in what’s not seen.”* (Co-Creation Interview, Participant #5)

Lastly, participants discussed how community engagement is often most successful when researchers have strong relationships with a community leader or community liaison; pastors are excellent touchpoints for sharing resources and educational materials:

*“… engaging the pastors and starting there. If you can get pastors excited about anything or if you can help steer the importance or… put things higher on a pastor’s priority list, you can bring numbers. Because one thing in communities of faith, they will follow their pastor…”* (Co-Creation Interview, Participant #4)

In other words, in some communities, engaging pastors may be useful for effective engagement with the community as a whole, and emphasizing inborn understandings of stewardship, intergenerational engagement, and eco-spirituality in religious communities.

#### 3.1.3. Inductive Analysis: Intergenerational Engagement Ensures Intergenerational Inclusion

All community leaders urged that truly effective community engagement and co-creation must be intergenerational. In our interviews, we only spoke with middle-aged to older adults; therefore, much of the discussion surrounding intergenerational engagement included concerns about their children, grandchildren, future kin, and the world we are leaving to the care of future generations. Some of this sentiment also came from a religious or spiritual standpoint (see 3.1.2). Participants told lively stories about their own experiences with intergenerational projects and the benefits that ensued:

*“But the kids learned from us, and we learned from them … We learned. We did different projects. It was so much fun … Not many schools do it and I think that’s a great way of getting kids involved in different issues and how the different generations see things and how they can come together as one and make a change.”* (Co-Creation Interview, Participant #6)

Therefore, participants’ experiences with intergenerational projects were often successful and fruitful. Participants also discussed tools and strategies for reaching folks of all ages, often citing social media as a useful tool to reach folks across generations:

*“So, I definitely realized through my experience, that social media has become something that every one of all generations are using because … when churches closed down and community centers closed down during the pandemic, that’s when social media became an intergenerational tool. Meaning prior to that, you would not see a 70- or 80-year-old person on social media, but now, that’s something that from the youngest person to an older person, you could actually use to engage in the community.”* (Co-Creation Interview, Participant #5)

Engagement involving all generations was therefore not even in question for the participants—it was a must-have for any research intervention. Intergenerational engagement allows for additional and needed layers of knowledge within research processes.

#### 3.1.4. Inductive Analysis: Human–Environment Relationships Vary by Individual and Community

This theme emerged from discussions about common environmental relationships and goals across communities, as well as the importance of recognizing individual- and community-level differences in environmental relationships. Firstly, participants agreed that a common goal across communities is to have a healthy, safe, and thriving community:

*“The commonality point where all people are together on that... Like everybody likes trees, everybody likes to have beauty in the community and have a place to feel safe and be recreational and enjoy the community. And I believe that this is the common point in the community.”* (Co-Creation Interview, Participant #2)

Multiple participants also mentioned how a relationship with the environment is important, especially for mental health and wellbeing. In particular, participants discussed how “watching things grow” ignites curiosity and emotional wellness within people of all ages.

That said, participants discussed how people have different relationships with the environment, and it is important to remember this while engaging so as not to take on a “one-size-fits-all” approach. Participants had varying individual levels of care for the environment, and recognized differences between their communities (i.e., a tree-lined block in Queens vs. a park-poor neighborhood in Manhattan). Participants also told a myriad of stories about the harms that come from the urban environment. Understanding individual- and community-level differences in relationship to the environment helps to understand both the benefits and harms:

*“One thing I had thought about as I was thinking awhile back about the trees and where you would plant these trees, there are trees that have become a living nightmare. And it was in the news the other day where the trees uproot the ground in front of people’s homes and it’s a tripping hazard. It was in the news. This one guy was complaining all the time… nobody ever did anything and he complained for years and people have gotten hurt.”* (Co-Creation Interview, Participant #6)

Some participants discussed a current lack of care in their community towards the environment, and that if human–environment relationships are mended with the help of researchers, community members can re-establish a sense of ownership and care with the urban environment:

*“So, they’re not going to grow overnight, but success would be people looking out for these trees, caring about their community… Don’t throw trash. Now they’re going to be more focused. They don’t want to see the trash. They don’t want to see any of this. So now they’re more watchful. They’re looking out for their own. It’s not the attitude, ‘Well, I don’t care. It’s not mine. I don’t own this, so what do I care?’ But it’s where you live. When they see that it makes a difference and how different it looks, how beautiful it can look, then they may be more inclined to maintain it.”* (Co-Creation Interview, Participant #6)

Overall, these points also emphasize the importance of attachment to place, and how different human–environment relationships may influence the care of a place, both relationships which researchers must understand to plan beneficial interventions.

#### 3.1.5. Inductive Analysis: Community Building Makes Co-Creation and Knowledge Sharing Between Communities and Researchers Possible

This theme emerged as foundational to all other themes. Participants spoke of community building as the creation of community and collaboration through events and meetings. It ensures the respective community’s human–environment relationships (spiritual, religious, cultural, or otherwise) are understood and uplifted. Community building was seen as the crux of effective, justice-centered, intergenerational community engagement. Participants discussed how building community requires time, partnerships with community leaders, and trust. In particular, building trust and community buy-in ensures that people feel comfortable enough to share their thoughts:

*“Well, my idea of trust is, where you feel comfortable with the person or groups that you are working with, that no one is going to feel that he or she is being used… that they have bought in, that they buy into what it is you are trying to do. And then understanding what you are doing, they feel comfortable in sharing their thoughts… I think that there’s a social level of comfortability, and there’s an intellectual level of comfortability, so--that you’re not trying to pull something over on me, you know what I mean, that it’s good. This is for us.”* (Co-Creation Interview, Participant #1)

Community building should therefore be catered to the audience of the community messaging, event, or meeting. Participants gave examples for how to involve the schools and the senior centers in community building (e.g., intergenerational education [Results 3.1.3 Participant #6]), how to establish community touchpoints (e.g., community boards, community liaisons, and pastors [Results 3.1.2 Participant #4]), and how to build community through messaging and engagement (e.g., social media [Results 3.1.3 Participant #5]).

Because each community is unique and may have different relationships with the environment—whether due to historical injustices, cultural representations, or age demographics—participants urged that community engagement and community building should vary across communities. For many communities, most participants mentioned education as a tool for community building, particularly when it comes to taking care of the environment and community:

*“It’s just education, education, and I think… the more knowledge you [community] have, or the wisdom that you have been able to gain from this… that you [researchers] can implement in a way that is only beneficial, and this is what I see, the word, beneficial, to our community.”* (Co-Creation Interview, Participant #1)

Participants discussed how educational events can contribute to community building, inform people of all ages, and, in turn, provide opportunities for researchers to learn about community values.

### 3.2. Deductive Coding Analysis of Other Interviews

Using the themes outlined in Table 2 and Section 3.1, we deductively coded ten interviews, five from interviews with urban tree professionals (Urban Forestry Interviews), and five from interviews with additional community leaders (Community Interviews). The following similarities and differences arose for each theme (see Table 3 for a summary).

#### 3.2.1. Deductive Analysis: Environmental Justice and Health Equity Are Interlinked and at the Heart of This Research

Community leaders often professed the most frequent and direct experiences between environmental justice and health/well-being outcomes (e.g., asthma due to air pollution and greening for mental health). When links between health and environmental justice were made by Urban Forestry Interview participants, conversations were more solution-oriented. For example, urban tree professionals described how tree canopy and heat inequity can be solved with tree canopy goals (i.e., increasing tree canopy as a percentage of existing land in cities). Differently, Community Interviews utilized more narrative forms of their lived experiences and storytelling to describe examples of environmental injustice and health inequity. Nonetheless, solution-based thinking was noted among the community leaders through metrics of success related to underlying structural issues of power and dominance. For example, Community Interview participants focused on the reclamation of land as a solution to environmental injustice and health inequity:

*“We understand that land has been used to displace, subjugate, oppress, all sorts of other things… since the beginning, right? And as much as we don’t necessarily believe in ownership of land, we do think that sort of community ownership is key to self-determination.”* (Community Interview, Participant #4)

Thus, conceptualizations of solutions differed between urban tree professionals and community groups. Community leaders often spoke with passion about the need for community knowledge and experience to be embedded in the research process (procedural justice). Not only does this ensure the inclusion of all members of a community, but it also may produce the best outcomes due to intergenerational embodied knowledge:

*“I feel like for me, it’s just being humble and being in a listening mode and being open to learning, because there’s a narrative that like, ‘people probably don’t care about the environment.’ But in many ways, we were the original stewards of the earth…Like so much of why we were able to have the fruitful crops in this country was because of knowledge that came with enslaved Africans that they brought to bear on growing crops here. So I just feel like the information is there and the people, if we’re not imposing on them, then they’ll tell us what we need to know. Or they’ll demonstrate it. They’ll just, they’ll show us.”* (Community Interview, Participant #5)

That said, the uplifting of embodied knowledge was not only noticed by the community leaders. One participant in the Urban Forestry Interviews discussed the embodied knowledge and skill in communities that often goes unnoticed:

*“And this… is something that’s really undervalued in the literature is that there can be a lot of expertise… there were some communities that had a lot of people who worked in landscaping, or that kind of space. And so they knew a ton about how to manage trees and how to manage green spaces. And so they would just do things themselves. Sometimes they got in trouble with the city. But you can do that. And that helps as well, that’s an extra layer of labor that’s going in that comes from their embodied expertise.” *(Urban Forestry Interview, Participant #3)

This was the only mention of embodied knowledge in Urban Forestry Interviews, though, so this concept was much more emphasized in the other community conversations. However, all participants in all three interview types recognized environmental injustice (e.g., heavy air pollution and lack of green space) and the historic and systematic drivers of injustice (e.g., redlining). Participants across the board recognized the importance of listening to community lived experiences, and engaging and educating communities.

#### 3.2.2. Deductive Analysis: Religious and Spiritual Communities Are Important Touchpoints in Urban Greening Research

There was no notable mention of environment, faith, and spirituality in either the Urban Forestry and Community Interviews.

#### 3.2.3. Deductive Analysis: Intergenerational Engagement Ensures Intergenerational Inclusion

Participants from all interview types discussed the crucial knowledge within community members of all ages, emphasizing the important of intergenerational engagement.

When discussing intergenerational engagement, the participants in the community-based interviews focused on storytelling, social media, and specific types of intergenerational events. Community participants described in-depth what intergenerational engagement looks like, sounds like, and feels like. For example, one participant connected intergenerational engagement with the reclamation of ancestral and indigenous knowledge:

*“I think also what is emerging for me as like, things unfold, is wanting to ground my work in this idea of healing and reclaiming and remembering indigeneity, like, indigenous knowledge, or remembering the ways that elders have held spaces or created ceremony, etc., that those things are also important to communicate to younger people.”* (Community Interview, Participant #5)

Intergenerational engagement therefore not only emphasizes the importance of engaging the youth and the future stewards of the earth, but also passes on the embodied knowledge of elders in the past and present.

#### 3.2.4. Deductive Analysis: Human–Environment Relationships Vary by Individual and Community and Community Building Makes Co-Creation and Knowledge Sharing Between Communities and Researchers Possible

Participants across all interview types agreed on the importance of a healthy relationship to trees and the natural environment. Participants in all interviews also discussed solutions for understanding these relationships through community engagement and community building, particularly through educational events and materials.

Community leaders focused more on community building and education as a necessity for mental wellbeing and community cohesion. Participants in the Urban Forestry Interviews, on the other hand, were more focused on community stewardship and capacity-building (e.g., green career development and robust volunteer tree stewardship programs) as a benefit from improved attachment to place, highlighting the relationship as a means to an important end:

*“I think we have some very clear linkages and I guess the arrow really goes two ways, between place attachment and stewardship actions…just talking to people about why they engage in caretaking of their place, whether it is trees or gardens or parks or whatever… having that deep sense of place and place attachment is a real driver for taking actions of care.”* (Urban Forestry Interview, Participant #4)

Urban Forestry participants were almost always limited in their tree care and maintenance funding and resources, particularly for communities already lacking quality tree canopy and the resources to improve tree maintenance. Sometimes, community attachment to place and subsequent stewardship was a necessity to ensure trees receive the timely care they need.

While community leaders also recognized the importance of caring for a place, they focused less on stewardship as a goal for communities, but rather how education can help foster human–environment relationships for the purposes of community building and improved mental health. Compared to the Urban Forestry Interview participants, the community leaders also more often recognized the harms brought on by urban greening, including green gentrification, as well as the competing interests that community members may rightfully have before the urban environment (e.g., financial concerns, childcare concerns, employment concerns, and other daily stressors).

### 3.3. Results of Member Checking and Research Co-Creation

In January 2025, we met with five of our six participants from the Co-Creation Interviews to institute member checking to this study and update members on the research findings. Co-Creation participants agreed with our findings across the five themes, and at times anticipated some of the themes prior to our description. Participants were also eager to share additional stories that related to their points (e.g., past experience in planting trees). The additional notes are summarized below:

Community participants in member-checking meetings stressed the important connection between economics as a determinant of environmental justice and health equity, especially since many heat-vulnerable communities have experienced historic economic underinvestment. When discussing religion and spirituality, participants expanded this theme beyond Christianity and Abrahamic religions; in particular, they mentioned Indigenous American belief systems and other spiritual connections that recognize the Earth, water, and sky as sacred beings, encouraging reciprocity of care. Participants also emphasized that engagement events should focus on understanding the priorities of community members, such as other health concerns, financial concerns, and immigration concerns. Communities that currently experience inequities in the basic social determinants of health (e.g., economic, education, and environment) will require an extra effort when it comes to community engagement. Participants provided further examples of community building through online sources as well as in-person events. Of mention was encouraging the “adoption” of trees by community members, which would not only allow them to be part of the planning process but also enable them to name each tree after community leaders and activists. This was in line with community participants’ further emphasis of intergenerational engagement and including older generations to facilitate the passing on of knowledge and legacies.

## 4. Discussion

### 4.1. Discussion of Emergent Themes

Emphasizing the importance of community engagement and the procedural aspects of environmental justice, our research team embarked on a co-creation process to better inform the use of urban greening as a heat-adaptive tool in cities. We conducted and compared three interview types: Co-Creation Interviews, Community Interviews, and Urban Forestry Interviews. The Co-Creation Interviews were conducted to guide the establishment of community co-creation and engagement throughout our research process. Five themes emerged and were compared with the Community and Urban Forestry Interviews to facilitate multistakeholder solutions: (1) environmental justice and health equity are interlinked and at the heart of this research; (2) religious and spiritual communities are important touchpoints in urban greening research; (3) intergenerational engagement ensures intergenerational inclusion; (4) human–environment relationships vary by individual and community; and (5) community building makes co-creation and knowledge sharing between communities and researchers possible.

Each theme aligns with well-established theories and concepts within the literature. Therefore, theories within the literature parallel the embodied knowledge within engaged communities; many themes from peer-reviewed literature sources were already known and articulated by community leaders (e.g., intergenerational engagement, environmental justice as a social determinant of health, intersectionality, and religion/spirituality and the environment) [41,55,56,57]. Participatory planning methods that uplift the embodied knowledge of place can inform urban design and provide experiential knowledge that may otherwise be overlooked [43]. Transdisciplinary research approaches encourage the inclusion of these factors through meaningful community involvement, which is imperative for the multidisciplinary and complex urban greening solutions needed to address climate change [28,38]. In particular, we saw the importance of public health considerations in community engagement (see Results Section 3.1.1 and Section 3.2.1). Previous urban greening research has included similar co-creation and procedural justice tactics, including participatory planning, participatory spatial modeling, and multistakeholder workshops [34,58,59]. However, few studies have considered community co-creation in the context of both heat-related health outcomes and urban greening [45], ensuring procedural justice is upheld in multidisciplinary endeavors as well.

### 4.2. Comparing Co-Creation Interviews and Other Interviews

These themes have and will continue to inform our research practice, but they also illuminate important opportunities for future exploration from interviews with urban forest professionals. Firstly, differences between interviews were noted in the various ways of producing knowledge. For example, in the Community Interviews, experiential and cultural knowledge was often shared, often with storytelling or anecdotal evidence. In contrast, the Urban Forestry Interviews contained more problem-solving language, and the pursuit of scientific evidence-based solutions. While both types of knowledge are important, our Co-Creation Interviews and Community Interviews emphasize experiential, Indigenous, and embodied knowledge, which is often overlooked in Western science [60]. For researchers entrenched in the world of empirical knowledge, upholding experiential and embodied knowledge is easier said than done; to do so requires challenging current power structures [61]. That said, part of the solutions to the problems that cities are trying to address (e.g., heat inequity, health inequity, and environmental injustice) may be found among communities experiencing these circumstances. In our case, combatting heat-related health issues through community-centered urban greening, we found that solutions to community engagement were often better articulated through highly practical advice and experiential examples from community participants. Other studies that have implemented community co-creation and participatory processes in urban green space projects have similarly found increased quality of analysis, validation of modeling, and a more sophisticated understanding of place-based variables [34,58].

Another opportunity to further explore in the differences between interviews lies in the scale of the problems faced by urban tree professionals and community leaders. Often, urban tree professionals were extremely passionate about their work to improve tree canopy cover and tree equity, focusing on accomplishing goals (e.g., securing more funding for maintenance, bolstering community engagement and stewardship, increasing a city’s tree canopy cover, etc.). Yet, how and to what degree urban tree professionals interact with urban environments differs fundamentally from community members who reside and experience the positive and negative impacts of urban greening far more intimately and ongoingly. Community-based metrics of success therefore first consider what it is like to experience and live with a tree-lined street, and the holistic benefits and harms that come, rather than what it means to achieve a certain percentage of tree canopy coverage, per se.

Finally, there was considerably more emphasis on community capacity building for tree stewardship in the Urban Forestry Interviews. This may be explained by the nature of the participants– the urban tree professional’s role and passion is to expand tree cover and maintain existing canopies, often on public land. Every urban tree professional we spoke with mentioned a lack of funding and resources for effective urban tree maintenance of private and public trees across the urban forest, much less community engagement. Communities that steward their own neighborhoods lessen the burden of tree maintenance for urban forestry departments that are already stretched thin [62]. That said, unfortunately, often the communities with the most maintenance issues are the ones that have less capacity to steward their own trees (e.g., due to financial constraints, time constraints, health constraints, etc.), particularly when trees are on private property and stewardship is volunteer-based [63]. Sometimes the burden of “stewardship” is therefore thrust upon those who lack the resources to do so in the first place.

### 4.3. Reflection on the Co-Creation Process

The co-creation process has been fruitful and rewarding on both sides. It has allowed us as academic researchers to challenge the traditional timing of research, as well as the traditional hierarchies of knowledge, holding community knowledge at the same level as our own. The community co-authors similarly emphasized their excitement for the research process thus far, and a newfound appreciation for urban greening and public health research.

Through this process, the emergent themes have operationalized practical tactics conducive to multistakeholder co-creation, including the following: 1. prioritizing in-person meetings whenever possible and preferred by the community participant; 2. attending and presenting at additional events/venues to build relationships and community (e.g., health fairs, participants’ workplaces, community boards, and educational workshops); 3. strengthening online resources (e.g., websites and social media); 4. incorporating intergenerational engagement during research/events; and 5. focusing less on capacity for tree stewardship, and rather on more prominent concerns/capacity-building needs based on specific communities.

We have since built key community partnerships in-person, which has allowed us to see the local trees near participants, as well as the places community members live, work, play, and pray. Understanding the local context of a community will eventually facilitate the integration of community concerns into future greening designs (i.e., understanding the need to avoid uplifted roots in neighborhoods with accessibility concerns). Additionally, we started this research with no online presence, and our conversations have since taught us that social media and other online resources are often the best methods for intergenerational engagement and communication; therefore, the creation of our own website is now underway.

### 4.4. Limitations and Future Work

This study is limited in that our deductive coding was done with interviews with different interview questions; therefore, a baseline level of difference is automatically expected between responses. That said, our intention was not to experimentally assess differences between urban forest stakeholders, but rather to explore common themes touched upon across conversations. Another limitation is that our study utilized a snowball sampling technique, which may have selectively sampled individuals with similar perspectives. However, we made efforts to intentionally interview snowball-sampled participants from different geographic areas or with different occupations to widen perspectives. Additionally, our study only analyzed a small sample of the Community and Urban Forestry Interviews, indicating that saturation for these interviews has yet to be reached. Furthermore, our study engaged primarily NYC-based community members, another limitation that may impact generalizability for the conclusions. Nevertheless, we expect these interviews to mark the beginning of our community engagement process, which we expect to change over time as we continue to engage communities, and which will also inevitably increase the sample size. Inherently, community engagement is an iterative process, and our methodology is sensitive to this. Thus, our research intends to guide researchers on the process of co-creation rather than solely the conclusions (e.g., emergent themes), all of which may vary across different communities both geographically and temporally. Furthermore, we recognize that NYC is one of the most diverse cities in the world, and this work serves as a starting point to consider diverse community perspectives beyond NYC. However, as it stands, this study can still help to inform researchers in smaller cities or different regions on how they can approach their co-creative research process.

In particular, national and local agendas and policies will benefit from process-based research on co-creative practices. For example, the US 2024 National Heat Strategy recounts urban green space as a key infrastructure strategy to combat extreme heat, but with vague instruction how to do so effectively [64]. Additionally, as mentioned in the Introduction, urban forestry is growing in popularity, as evidenced by recent national funding (i.e., through US Inflation Reduction Act funds), and local interest (i.e., Local Law 148 in NYC codifying an Urban Forest Plan). The formation of the NYC Urban Forest Plan is currently underway and has similar goals of multistakeholder, participatory, community-engaged planning, as evidenced by a host of community events and workshops influencing the plan [65].

This work therefore informs current urban forest plans and our ongoing research practice towards transdisciplinary and community-engaged scholarship. Our future research will focus on modeling transdisciplinary heat-adaptive solutions, so we look forward to expanding co-creation beyond qualitative research into quantitative modeling, particularly as we keep in mind additional concerns communities have raised aside from heat (e.g., asthma, lack of healthcare access, and financial concerns). These tactics will allow us to create urban greening solutions that are meaningful to the communities we aim to protect.

Future research should also further elucidate the challenges and opportunities between urban tree professionals, community leaders, and other urban forest stakeholders through combined conversations (i.e., focus groups). Future questions to explore can include assessing tensions between experiential and scientific knowledge, and exploring stakeholder differences in preferences and practicalities for community co-creation. Finally, as emphasized by our participants and the importance of intergenerational engagement, future research should involve adolescents through interviews and focus groups as well.

## 5. Conclusions

The heat-related impacts of the climate are worsening, putting additional risk on those most vulnerable (e.g., older adults, young children, minorities, low-income individuals, etc.). As researchers looking to address climate-induced health inequity through scientific methods and transdisciplinary collaboration to produce tree planting heat-adaptive scenarios, we also recognize the community-based implications of implementation.

Our conversations with community leaders in NYC and urban tree professionals across the US help to operationalize the pre-established theories that naturally emerged from conversations with our community partners. We are currently working on building communities through in-person meetings and events, educating and disseminating information online, and considering community-specific concerns both for our future research and for community presentations. Moving forward, we also anticipate exploring additional ways for community members to be involved in the research model validation, data analysis, and dissemination, as well as to further explore opportunities and challenges between stakeholders. Determining what this continuous engagement looks like will be built by similar co-creation tactics and multistakeholder collaboration such as what has been done for this particular study, as well as through adopting previous co-creation/participatory approaches found in the literature. For now, we will operationalize the community engagement tactics as described in Discussion Section 4.3.

This paper marks the beginning of our research team’s community co-creation process, and we look forward to utilizing additional co-creation methods as the research continues. The future of cities will inevitably include urban forestry; we hope this research further encourages equitable urban greening practices, so our urban trees provide the health benefits as intended for years to come.

## Figures and Tables

**Table 1:** Descriptions of interview types and participants for this manuscript.

Interview Type	Description	Number of Participants	Number of Interviews	Location
Co-Creation Interviews	Interviews that addressed how we can establish community co-creation and engagement throughout our research process. Questions for the Community Interviews were workshopped in these meetings as well	6	6	Community leaders in Harlem, New York City
Urban Forestry Interviews	Interviews that explore the abstract and practical considerations of tree planting and management related to cooling	10	5	Urban tree professionals in the contiguous United States (Chicago, IL, USA; Durham, NC, USA; Los Angeles, CA, USA; New York, NY, USA; Philadelphia, PA, USA)
Community Interviews	Interviews that explore community perceptions around trees and urban greening as a solution to heat	5	5	Community leaders in heat-vulnerable neighborhoods in New York City (Chinatown, Manhattan; Flatlands, Brooklyn; Harlem, Manhattan; South Bronx)

**Table 2:** Emergent themes from Co-Creation Interviews (inductive coding).

Theme	Description
Environmental justice and health equity are interlinked and at the heart of this research	Link between environmental justice and health equity Environmental injustice and health inequity affect medically underserved communities (i.e., low-income communities of color) Procedural justice is imperative (co-creation, intergenerational community engagement, transparency, and education) Communities deserve evidence-based information about benefits from research and interventions
Religious and spiritual communities are important touchpoints in urban greening research	Faith communities feel a responsibility to be stewards over the world (God’s creation), and stewardship is rewarded with environmental benefits Faith is believing in what cannot be seen—just like the nurturing of a seed to a fully grown tree Pastors can be helpful touchpoints to reach faith communities
Intergenerational engagement ensures intergenerational inclusion	Other generations want to leave behind a better world for their children and future kin Storytelling and social media are important intergenerational tools Individuals of all ages have different, and equally important, types of knowledge
Human–environment relationships vary by individual and community	Everyone wants a healthy, beautiful community People have different relationships with the environment depending on their background and life experiences It is important to consider the ways in which the environment harms communities Planting and caring for something and seeing it grow is good for mental health Strong relationships with the environment emphasize the importance of community ownership and care for the environment
Community building makes co-creation and knowledge sharing between communities and researchers possible	Community building ensures community values are understood and justice-centered plans are implemented Community building happens through intergenerational engagement that involves trust and education Community building often requires relationships with community leaders (e.g., community board members and pastors)

**Table 3:** Similarities and differences in urban tree professional and Community Interviews (deductive coding).

Theme	Similarities	Differences
Environmental justice and health equity are interlinked and at the heart of this research	Recognized the historic and systematic drivers of injustice Recognized the importance of listening to community lived experiences, engaging and educating communities	Community leaders emphasized the links between environmental justice and health outcomes/equity (e.g., asthma due to air pollution) Community leaders were more focused on the reclamation of land and that solutions already exist in the embodied knowledge within communities While community leaders emphasized storytelling and experiential knowledge, urban tree professionals focused on metrics of environmental justice
Religious and spiritual communities are important touchpoints in urban greening research	Faith mentioned in the context of individual faith and how faith guides goals of caring for others and the earth	There was no mention of environmental faith and spirituality in both the Urban Forestry and Community Interviews
Intergenerational engagement ensures intergenerational inclusion	Discussed the embodied knowledge within communities and people of all ages	Community leaders had more emphasis on intergenerational storytelling, social media as an intergenerational tool, and specific types of intergenerational engagement
Human–environment relationships vary by individual and community	Emphasized the importance of attachment to place with the natural environment	Urban tree professionals were more focused on volunteer community stewardship Community leaders emphasized how different people have different feelings about the environment Community leaders emphasized ways in which education can be a part of fostering human–environment relationships
Community building makes co-creation and knowledge sharing between communities and researchers possible	Discussed solutions for community engagement and the importance of community building for capacity building Emphasized benefits of educating communities	Urban tree professionals were more focused on how to build capacity and empower community members to help foster stewardship Community leaders were more focused on mental health benefits and empowerment of community building

## Data Availability

The datasets presented in this article, including anonymized transcripts, are not readily available because the data are part of confidential interviews.

## Supplementary Materials

The following supporting information can be downloaded at https://www.mdpi.com/article/10.3390/ijerph22060896/s1: Supplementary File S1: Interview schedules.

## Abbreviations

The following abbreviations are used in this manuscript:

CAB	Community Advisory Board
NYC	New York City
