# Supplementary material for: Exploring Community Co-Creation in Tree Planting and Heat-Related Health Interventions: A Qualitative Study
Source: Int J Environ Res Public Health. 2025 Jun 4;22(6):896. doi: 10.3390/ijerph22060896 (PMC12193202; doi:10.3390/ijerph22060896)
Supplement: Supplementary file 1 [file ijerph-22-00896-s001.zip › ijerph-3592903-supplementary.pdf]

Supplementary File S1: Interview Schedules

1. **Co-creation Interviews**

Questions about Co-Creating the Research Design

Co-creating research ensures voices of participants are heard and valued to the same extent as scientific knowledge. For us, this looks like building questions for the interviews and focus groups together, communicating about how we can analyze data from interviews and focus groups, and working together on presenting our findings. That being said, we want to hear about the best ways to work together for co-creating the research design. The questions below will guide us throughout the research process.

Before Engaging with Communities	• How can we creatively engage with communities beyond just focus groups and interviews? (i.e., should we give an educational presentation at a church, school, community garden; should we help facilitate other tree-focused events such as through social media presence or community art installations?)<br>• How can we balance involving community members in our research design while also being respectful of community members' time?<br>• How can we incorporate intergenerational knowledge and experience into our engagement?<br>• What is the best time of year to interview the folks in your community?
Interviews	• What is the best time of year to interview the folks in your community?<br>• Are the interview questions a good way to ask about trees in your community? Are there better ways/other considerations we should have?
Analysis of Interviews	• How should results be interpreted? How do we involve community members in the analysis?<br>• Does it make sense to include a community member in the analysis process? What is the going rate for compensation?<br>• If we were to include a community member in analysis, would a local university student be an appropriate option?
Delivery of results	• How do we involve community members in the analysis and manuscript writing?<br>• What is the best way for the results from interviews and focus groups to be presented? How can we incorporate community member's

	desires into publications, presentations, other events?<br>• How can we be creative with presentation of results? Creative examples include:<br>o Proposing visual scenarios of tree planting<br>o Drawing ideal tree planting scenarios in your community<br>o Providing maps to demonstrate locations to plant trees
Questions Asked Throughout the Research Process	• What are you all interested in learning from us throughout the research process?<br>• What voices in your community have we not considered?<br>• **How are we going to measure success with community members? (What are outputs we want to have?) see below.**

- **How are we going to measure success with community members?**

**Short-term**	
**Mid-term**	
**Long-term**	

- **How are we going to measure success for our team?**

**Short-term**	• Establish good connections with community members and organizations<br>• Focus groups on the co-creation of the research show positive feedback
**Mid-term**	• Publish papers about co-creating research design with help from community
**Long-term**	• Trees are planted in the places we've worked in<br>• We have a relationship with NYC Council and the advisory board for the 1065 Master Tree Plan

2. Community Interviews

Interview Schedule

Introduce ourselves and our research on trees, heat, and the community

Introduction to You and Your Community

- Tell us a bit about yourself.
- Tell us about your neighborhood.
- Who is a part of your neighborhood?
- Who do you represent?
- Most people feel like they belong to a community (or communities). How would you describe your community?
- What does the term "urban greening" mean to you?
- What does urban greening look like in your neighborhood today?
- How has urban greening changed in your neighborhood since you've lived there?
- Who maintains the green space in your neighborhood?
- Can you tell us about any experiences you've had with tree professionals?

Questions Related to Trees In General

- How do you feel about trees?
- Trees can be harmful or helpful. How have you seen that in your neighborhood?
  - Can you give any examples?
- How does your opinion reflect the ways your community feels about trees? Does this differ from your neighbors and community? Why or why not?
- Can you describe if and how trees relate to your health?

Questions Related to Trees and Heat Risk

- The summers are getting hotter. What do you do to keep cool in the summer?

- What do you know about trees and their ability to cool?
    - Do you think this is important? Why or why not?

- How do trees fit into your cooling strategies, if at all?

- If trees really do cool a lot, would that change your opinion of them, especially as summers get hotter?

Conclusion

- There is a big campaign to plant more trees around New York City. For you, your neighborhood, and your community, what does that mean? Is that a good thing? What does success look like?

Do you have any questions for us? Any other thoughts you would like to share?

3. Urban Forestry Interviews

<u>Introduction</u>

Thank you for meeting with us. Our team is studying how trees could help to protect urban communities from climate change-amplified extreme heat events. Our motivation is that a warming climate will increase heat waves, risk of heat-related illnesses, emergency department visits, and even deaths. We think that urban forestry practices could help protect urban communities, and particularly their most vulnerable members.

Our broader project focuses on quantitative models of the effects of trees on heat exposure and the associated heat-related emergency department visits. Specifically, we are curious about how urban forestry professionals think about the health implications of their work. We are also curious about the abstract and practical concerns urban forestry professionals have about using trees to reduce heat. This could include current or future challenges that are political, economic, or logistical. We would like to spend some time discussing your perspectives on this topic.

To help us as researchers, we would love to take notes as well as record this interview so we can carefully review it. Is that okay? As part of the research process, if you agree to be interviewed, we also ask that you sign a consent form.

<u>Introductory Questions (5 min)</u>

- Describe your current role, and your previous experiences.
- What other stakeholders do you engage with as a part of your role?
- What policies impact your role? What laws impact your role?

<u>1. Our project: Scientific Basis of Tree Cooling in Extreme Heat Events (10 min)</u>

- Our study hypothesizes that specific tree species vary with their degree of cooling. We've heard from multiple folks that trees with bigger leaves or a bigger overall crown

most likely have better cooling effects, rather than specific species type. What are your thoughts about how specific trees species vary in their cooling effects?
- How does your urban forestry program make their tree selection decisions? To what extent do you make tree selection decisions based on trees' cooling effects? What would it take for you to use ecosystem services as a factor in tree species selection?

3. Tree Selection and Planting (15 min)

- We are somewhat familiar with the process of tree selection and planting for other urban forestry programs. Can you speak more to how the tree species selection decisions are made?
    - Can you speak towards how climate change and climate resilience affects these decisions?
- We are finding in interviews that tree survivability is more valuable in species selection decisions than ecosystem services/health benefits provided. How do you weigh different ecosystem services in tree species selection, if at all?
- How can a tree species recommendations based on ecosystem services (i.e., cooling benefits) be helpful for urban foresters?

4. Tree Management/Maintenance and Stewardship (10 min)

- What are the main challenges you are seeing with maintenance?
- What are the lessons you've learned in relation to planting and maintenance for the future? What challenges still exist?
- In an ideal world, what would a "perfect" maintenance plan look like for urban trees? (What types of funding, what types of equity considerations/community engagement)
- What are your thoughts about stewardship of trees as it relates to the maintenance and care of them?

5. Stakeholders, and particularly communities (10 min)

- We've heard from some folks that sharing the public health benefits of trees may be an effective way to garner community and governmental support for trees. What do you think about this? Do you have any other ideas for the best ways to gain support for tree programs?
- On a similar vein, we are finding that community members value educational programs and materials to learn about the urban forest and engage with urban forestry. What are some ways your program is already doing this and in what ways do you think more can be done?
- We've also heard about various barriers to implementing an equitable tree planting/maintenance plan. What do you see as the main barriers to equitable tree cover in the city?
- How can we work towards improving tree equity moving forward?

IF TIME or if it comes up naturally: Ecosystem Services and Health (10 min)

- We've asked previous interviewees to rank the ecosystem services of the urban forest by importance. Generally, we've heard a lot about cooling, stormwater, air quality, cultural/mental/spiritual benefits, and biodiversity benefits. Of these

ecosystem services, or others I didn’t list, which do you see as the most important or relevant to your work?
- We’ve generally heard from other urban foresters that they do see the links between their role and public health, but often they do not work closely with public health practitioners. What are your thoughts on this – in what ways are you working with public health folks or in what ways do you wish you were working with them?

6.Final thoughts (2 min)

- Do you have anything else you want to say?
- Do you have any questions for us?
